# Migratory New World Blackbirds (Icterids) Are More Neophobic than Closely Related Resident Icterids

**DOI:** 10.1371/journal.pone.0057565

**Published:** 2013-02-27

**Authors:** Claudia Mettke-Hofmann, Hans Winkler, Paul B. Hamel, Russell Greenberg

**Affiliations:** 1 School of Natural Sciences & Psychology, Liverpool John Moores University, Liverpool, United Kingdom; 2 Smithsonian Migratory Bird Center, National Zoological Park, Washington, D. C., United States of America; 3 Konrad-Lorenz-Institut of Ethology, Department of Integrative Biology and Evolution, University of Veterinary Medicine, Vienna, Austria; 4 Southern Hardwood Laboratory, U. S. Forest Service, Stoneville, Mississippi, United States of America; Université de Strasbourg, France

## Abstract

Environments undergo short-term and long-term changes due to natural or human-induced events. Animals differ in their ability to cope with such changes which can be related to their ecology. Changes in the environment often elicit avoidance reactions (neophobia) which protect animals from dangerous situations but can also inhibit exploration and familiarization with novel situations and thus, learning about new resources. Studies investigating the relationship between a species’ ecology and its neophobia have so far been restricted to comparing only a few species and mainly in captivity. The current study investigated neophobia reactions to experimentally-induced changes in the natural environment of six closely-related blackbird species (Icteridae), including two species represented by two distinct populations. For analyses, neophobic reactions (difference in number of birds feeding and time spent feeding with and without novel objects) were related to several measures of ecological plasticity and the migratory strategy (resident or migratory) of the population. Phylogenetic relationships were incorporated into the analysis. The degree of neophobia was related to migratory strategy with migrants expressing much higher neophobia (fewer birds feeding and for a shorter time with objects present) than residents. Furthermore, neophobia showed a relationship to diet breadth with fewer individuals of diet generalists than specialists returning when objects were present supporting the dangerous niche hypothesis. Residents may have evolved lower neophobia as costs of missing out on opportunities may be higher for residents than migrants as the former are restricted to a smaller area. Lower neophobia allows them approaching changes in the environment (e.g. novel objects) quickly, thereby securing access to resources. Additionally, residents have a greater familiarity with similar situations in the area than migrants and the latter may, therefore, initially stay behind resident species.

## Introduction

Environments change predictably and unpredictably due to seasonal and catastrophic events, global warming and human impact. Species have evolved adaptive responses to uncertainties such as changing habitats, occurrence of new resources, or new competitors. Such changes usually elicit initial avoidance (neophobia) which protects individuals from encountering dangerous situations [1], [2]. However, neophobia also delays getting in contact with valuable resources such as new food [3] (though the actual inclusion of a new food item in the diet is more influenced by dietary conservatism [4] than neophobia) and has been shown to delay learning [5], [6] and problem-solving [7]. It has also been identified as part of a larger complex of correlated behaviors known as behavioral syndromes or personality traits [8], [9]. Neophobia is known to differ between species (e.g. [10], [2], [6]) but despite the wide-ranging consequences of neophobia only few studies have ever investigated the underlying factors determining neophobia.

Ecological plasticity (measured as behavioral flexibility) has been proposed to relate to neophobia [10], [2]. For example, feeding generalists hesitate less to approach and feed from familiar food when a novel object is placed beside it than closely related feeding specialists [10], [11]. Similar results were found for habitat generalists and specialists [12]. All study subjects in these experiments were either wild-caught birds or birds tested in the wild, i.e., neophobia may have differed because of the different environments experienced (functional neophobia [13]). The results were explained with the neophobia threshold hypothesis [12] which states that experience collected early in life is protected by neophobia later in life. This means that the fewer experiences a young bird collects (e.g. because it is specialized on only one habitat type) the stronger neophobic reactions will be during adulthood. Thus, neophobia may prevent adult birds from becoming familiar with novel situations and favor ecological specialization [12]. However, other studies have found that in some species such as sparrows (*Melospiza*), [14] and ducks (*Anas*), [13] generalists are more neophobic than specialists. Other than in the studies mentioned above rearing environment was controlled in these experiments and intrinsic neophobia [13] measured. The results of these studies were explained with the dangerous niche hypothesis. It predicts that species that live in dangerous habitats or feed on potentially dangerous food should show high levels of neophobia to protect an individual from unknown potential danger of new things [13]. Generalist species, particularly those living in close proximity to humans, are exposed to a variety of unfamiliar situations and are therefore, more likely to encounter dangerous situations such as unknown predators, persecution etc. As a consequence, generalists may be more neophobic than specialists [13]. The highly neophobic responses to unfamiliar situations in feeding generalists such as ravens (*Corvus corax;* [15]), house sparrows (*Passer domesticus*; [16]) and rats (*Rattus norvegicus;* [17]) as well as in habitat generalists such as the shiny cowbird (*Molothrus bonariensis*; [18]) are further examples supporting the dangerous niche hypothesis as all these species have been subjected to acute persecution. Except for the link between persecution and neophobia (dangerous niche hypothesis) there is currently more support for the neophobia threshold hypothesis, particularly when considering studies in the wild [13].

Reactions to changes in the environment have also been shown to be related to the migratory strategy of a species – being migratory or resident – with migratory garden warblers (*Sylvia borin*) showing much stronger avoidance (neophobia) when confronted with a novel object beside the familiar feeding dish than the closely related but resident Sardinian warbler (*S. melanocephala momus*; [19]). The study has been conducted with hand-reared individuals suggesting a genetic component of differences in neophobic reactions.

Up to now, ecological plasticity (generalist-specialist) and migratory strategy have never been considered together in relation to neophobia. Moreover, most studies attempting to relate neophobia to ecological plasticity or migration were conducted in captivity often comparing just two species and thus provide a weak test of the effects of any ecological correlates.

We investigated the relationship between ecological plasticity, migratory strategy and neophobia reactions in eight taxa of New World blackbirds (Icterids) in the wild to study how neophobia may operate under ecologically and socially realistic circumstances. New World blackbirds have a diverse range of diets and habitats ranging from feeding and habitat generalists to specialists and also differ in their migratory behavior from residency to migratoriness (e.g. [20], [21]). In winter, New World blackbirds form mixed species flocks which allowed comparing neophobia reactions directly between species. Six closely-related species of New World blackbirds two of them represented with a resident and a migratory population each were chosen for the study resulting in eight groups for comparisons. Neophobia was elicited by presenting novel objects around feeding locations established for this study (following [22]). Based on earlier results in the lab [19] we expected migratory birds to show more neophobia than residents. Furthermore, New World blackbirds are often considered as pest birds and have a long history of human persecution. In the studied species the degree of persecution was correlated with habitat breadth but not with any other measures of generalism (see below). We thus expected habitat generalists to be more neophobic than habitat specialists following the dangerous niche hypothesis [13].

## Materials and Methods

### Species and Study Sites

We studied six blackbird species at two different geographic locations (California and Mississippi, Table 1) from December 2003 to March 2005. Two species (red-winged blackbird (*Agelaius phoeniceus*) and Brewer’s blackbird (*Euphagus cyanocephalus*)) were tested in both California and Mississippi providing data for different populations and migratory behavior (see below) of the same species, thus resulting in an overall sample of eight populations. Following [23], red-winged blackbirds in California were considered as resident, whereas the population in Mississippi is known to consist of residents and migrants during winter [24]. In the Brewer’s blackbird, the population west of the Sierras is mostly sedentary, whereas birds east of the Continental Divide are much more migratory [25]. Brewer’s blackbirds sampled in California were therefore categorized as resident; whereas Brewer’s blackbirds sampled in Mississippi were categorized as migratory (the species does not breed in Mississippi).

**Table 1:** Study species, geographic locations and independent variables.

Species	site	locations	bm (g)	hb	db	dc	mis
*Euphagus cyanocephalus* Brewer’s blackbird	CA	4	64.0	3	4	93	R
*Euphagus cyanocephalus*	MS	3	65.2	3	4	93	M
*Euphagus carolinus*Rusty blackbird	MS	5	61.6	2	6	31	M
*Quiscalus quiscula*Common grackle	MS	10	95.2	3	5	85	R/M
*Molothrus ater ater*Brown-headed blackbird	MS	8	44.6	3	2	37	R/M
*Agelaius phoeniceus*Red-winged blackbird	CA	6	54.0	4	3	78	R
*Agelaius phoeniceus*	MS	10	63.2	4	3	78	R/M
*Agelaius tricolor*Tricolored blackbird	CA	3	56.5	2	2	79	R/M

bm: body mass; hb: habitat breadth, db: diet breadth, dc: diet change (high numbers indicate a large change in diet (high plasticity), small numbers a small change in diet (low plasticity)), mis: migratory strategy, CA: California, MS: Mississippi, R: resident, M: migratory, R/M: resident and migratory; explanation of the variables see text.

Experiments in California were carried out between mid-October and mid-December 2004 on and around the Point Reyes National Seashore National Park (37° 59′ 51′′ N, 122° 45′ 28" W) and private properties where resident Brewer’s blackbirds [25], resident red-winged blackbirds [23], and resident and migratory tricolored blackbirds (*Agelaius tricolor*) [26] occur in mixed-foraging flocks. Experiments in California were carried out at six different sites on open fields and meadows with a fence or shrubs nearby and/or surrounded by trees. Sites were separated by a minimum of 5 km. Adjacent farms had different species compositions that remained stable over the study period. Therefore, it was unlikely that we tested the same individual at different sites.

Experiments in Mississippi were conducted in December 2003 and mid December 2004 to early March 2005 in the Mississippi Delta region near Greenville (33^o^ 27.3′N, 91^o^ 2.1′W). Here, flocks of red-winged blackbirds, brown-headed cowbirds (*Molothrus ater*) and common grackles (*Quiscalus quiscula*) consist of resident breeding individuals and overwintering migratory individuals [27], [24], [21]. Additionally, migratory Brewer’s and rusty blackbirds (*Euphagus carolinus*) overwinter in this region but do not have breeding populations here [20], [28]. All five species can be found in mixed-species foraging flocks of icterids. Experiments were carried out at 10 different sites at least 10 km from each other on the Yazoo National Wildlife Refuge, Leroy Percy State Park, Delta Experimental Forest and private farmland. Most sites were on open fields with or without scrub or trees in the surroundings, one site was located in an open pecan orchard and two sites were in the forest interior. Capture and color banding after the experiments revealed re-sightings of banded individuals only at the site of banding.

### Phylogenetic Relationships

Closely-related species tend to behave similarly and may not represent completely independent data points. We, therefore, considered phylogenetic relationships among the species based on mitochondrial DNA [29] in our calculation. For the eastern and western populations of the red-winged blackbird and Brewer’s blackbird we used the distance of the mtDNA nucleotide sequences (available in the Gen Bank) between *Agelaius phoeniceus arctolegus* and *A. p. gubernator* as an estimate for phylogenetic relationships between populations within each of the two species.

### Experimental Procedure

The blackbirds were attracted to a 1.5×1.5 m feeding plot on the ground. During the non-breeding season, the species under investigation primarily feed on seeds except the rusty blackbird which feeds to a high extent on insects throughout the year [20]. Therefore a mixture of food, appropriate for granivorous and insectivorous species was provided by covering the feeding plot with equal amounts of cracked corn, sunflower seeds, whole oat, rye grass and rough rice and a custom-made eggfood (basic composition: 10 boiled and chopped up eggs mixed with 1 cup cracked corn and 1 cup corn meal). The overall amount of food provided (average 10 cups) was adjusted to the number of birds visiting the feeding plot so that enough food was available for approximately 4 hours. Fresh food was provided daily at the same time (before dawn except four sites in California where food was provided around noon) and on experimental days directly before the experiment.

Experiments started approximately after 12 days (+/−6 SE) when the birds regularly visited the feeding plot and species composition appeared to be stable. A neophobia experiment consisted of about 1 h data collection without objects (control trial 1), followed by 1 h with objects around the feeding plot (experimental trial) and another hour without objects (control trial 2) to control for feeding motivation. Neophobia was tested by placing four identical novel objects outside the feeding plot at a distance of 30 cm to the corners of the feeding plot. Overall, six artificial objects were tested at each site. Novel objects were selected to be distinct from what is generally experienced by blackbirds and sufficiently different from each other to minimize the effects of stimulus transfer. The use of different objects ensured that reaction to novelty was tested (the only salient stimulus among all objects) rather than reaction to specific object patterns. The objects were light red plastic pyramids (30×15 cm H(eight) ×W(idth)), light blue and yellow plastic butterflies (20×20×1 cm W×H×D(epth)), red and white plastic candy sticks (30×3 cm H×W), blue plastic sunflower windmills (30×5 cm H×W), violet and black aluminum garlands (30×8 cm H×W) and bright colored dusters (30×10 cm H×W). All objects were attached to a stick to keep them in place and guarantee the same height for all experiments. The order of presentation varied between sites in a way that each object occupied each position at least once. At two sites (one in California, one in Mississippi) unfavorable weather conditions and disturbance required repetition of some of the experiments because of extremely low numbers of birds visiting the feeding plot. In these additional tests brown beer bottles (20×6 cm H×W) oriented upside down, light violet beakers (15×9 cm H×W) and a green hosepipe bent to a ring (20×2 cm H×W) were used. During the pilot study in 2003 objects were milk bottles (30×8 cm H×W) and bottles connected with a rope.

Observations were done from a blind at least 15 m (in the forest) or 50 m (in open fields) away from the feeding plot depending on the landscape. The observer entered the blind before dawn (except for four locations in California where experiments were conducted in the afternoon). A second person added and removed the objects in the experimental situation. The following data were recorded continuously: 1.) order of arrival of the species in center (feeding plot), 2.) number of birds/species in center and whenever a species was not in center how many individuals were either in vicinity (an area 1 m around the center marked with twigs on the ground), in outer area (an area approximately 5 m around the center) or nearby (on trees, bushes or on the ground in an area approximately 10 m around the center). The number of birds in center (in vicinity etc.) was recorded whenever a change occurred. When large numbers of birds were present (often more than 100 birds were feeding simultaneously), abundance of different species was counted sequentially. After all species had been initially counted, counting started immediately again in the same species order. It should be noted that although sparrows, cardinals (*Cardinalis cardinalis*) and other smaller birds sometimes fed prior to the arrival of blackbirds, when blackbirds were present no other species visited the feeding plot.

One experiment was performed per day. Generally, experiments were done on no more than two consecutive days followed by a pause of on average 2.5 (+− 3.04 SE) days to avoid habituation to the procedure. We assume to have tested the same birds repeatedly at a given site but different ones at distinct sites (supported by the capture results mentioned above).

### Phylogenetic and Statistical Analyses

#### Behavioral parameters

The continuous data flow was divided into 15 second units assessing the highest number of simultaneously feeding individuals per species in center within each 15 second unit. This measure is conservative as it reflects how many different individuals were feeding at a time and avoids problems of summing up numbers when individuals cannot be identified. For analyses, we calculated the following two dependent variables for the first and second control trial and the experimental trial: 1) mean maximal number of individuals per species in center, i.e., we calculated for the duration a species was present in center how many different birds on average were feeding at a time. As the overall number of birds present around the feeding plot (for calculation of the overall number see below) did not differ between control and experimental trials (Wilcoxon Signed Ranks test: n = 9, z = −1.244, p = 0.214) our measure reflects the relative number of present birds visiting the center. 2) Mean time spent in center per species which was calculated as follows; we assumed the same individuals were feeding as long as numbers remained the same or increased which reflects observations during the experiment. We used this uninterrupted foraging bout of stable or increasing numbers of birds of the same species as the unit to estimate individual foraging times. For example, ten birds started foraging and group size increased over the next five 15-second units and then dropped then the first ten birds had a foraging time of 75 seconds. When two more birds joined in during the second 15-second unit they received a foraging length of 60 seconds etc. These data provided information about how many birds foraged for how long which allowed estimating average foraging durations for individuals. Means were first calculated for single experiments within sites, then for single sites and finally across all sites.

The number of birds feeding and time spent in center may be a function of the number of individuals per species present. During control trials most birds concentrated in the center. During experimental trials, however, many birds were sitting in surrounding trees, bushes or on the ground and the number of birds in center did not represent the number of birds present. We, therefore, assessed the overall number of birds present for each species and trial in the following way; we took the value of the distance category (center, vicinity etc.) with the highest number of birds per species present for each 15 second unit. For example, if two birds were feeding in center and twenty were in vicinity during one 15 second unit the latter value was used. From these data we calculated an overall abundance value for each species for the two control trials and the experimental trial. The variable was later used as an independent variable for the multiple regressions (see below).

#### Test for neophobia

In a first step we tested for the two dependent variables (number of birds in center and time spent in center) whether the novel objects elicited a neophobic response by using repeated ANOVA with trial type (control 1 and 2 and experimental) as within-factor and species as between-factor variable. For this analysis means for each site per species were used. The variable ‘number of birds’ was Log_10_ transformed to achieve normally distributed data and equality of variances. The variable ‘time spent in center’ was normally distributed.

#### Ecological plasticity, migratory behavior and neophobia

Secondly, to investigate the relationship between ecological plasticity, migratory behavior and neophobia, we assessed the following independent variables based on information derived from the ecological literature on the species [27], [20], [24], [21], [26], [28]: Four habitat types (following [30], [31]) were distinguished – open fields, open areas with trees, forest edges and forest. The number of different habitat types used by a species reflects habitat breadth (Table 1). Furthermore, we distinguished six food categories [30], [31] – insects, grass/herb seeds, tree seeds, fruits, flowers and vertebrates. The number of different food categories incorporated in a species’ diet reflects diet breadth (Table 1). Diet change was taken as another measure for feeding plasticity. During summer, blackbird species feed to a considerable amount on insects. This changes dramatically in winter, when many but not all switch to a diet consisting primarily of seeds. The ability to switch diets requires not only plasticity in searching and handling techniques but also adaptations in the digestive tract [32]. From the literature, we determined the percentage of insects in the diet in summer and winter. The change in diet was expressed as the percentage of insects in the winter diet as compared to the percentage of insects in the summer diet (set to 100%). Fourth, we distinguished three migratory strategies. A population was categorized as resident when the population under investigation consisted of individuals that were present in the area throughout the year (red-winged blackbird and Brewer’s blackbird in California). A population was categorized as resident/migratory when it consisted of resident and migratory individuals (red-winged blackbird, brown-headed cowbird and common grackle in Mississippi and tricolored blackbirds in California). The rusty blackbird and the Brewer’s blackbird in Mississippi were categorized as migratory because they have no breeding population within 1000 km of the study site. As the fifth variable, we included body mass (g) as energy demands differ between size classes which may influence neophobic reactions (Table 1). Separate data about body mass were available for western and eastern blackbird populations [25]. The overall abundance of birds around the feeding location was added to control for an effect of abundance on neophobia reactions (sixth variable). Finally, a variable for the two geographic locations (California and Mississippi) was included to test for a possible different reaction at the two locations.

As dependent variables we calculated the difference in number of individuals per species in center and time spent in center between control trial 1 and experimental trial. The difference was expressed as the percentage of performance during the experimental trial in relation to the first control trial (100 × value for experimental trial/value for control trial). Values could range below and above 100%, for example a value of 75% means that 25% fewer individuals were feeding when objects were present than when no objects were present. Theoretically, birds could also be attracted by the objects [33] resulting in values above 100%. For the number of birds in center, values for control trial 1 and 2 were correlated (Pearson’s corr. r = 0.97, p<0.001). We therefore only calculated the difference between the first control trial and the experimental trial. The time spent in center did not correlate between the two control trials (r = 0.5, p = 0.212) and we also calculated the difference between the experimental trial and the second control trial.

Analyses for the overall species comparison were twofold: First, we used species as independent data points in multiple regression analyses where selection of variables was restricted to a maximum of two out of seven predictors (see below); secondly, we considered phylogenetic relationships among the species through the use of phylogenetic generalized linear models (PGLM; [34], [35]). In the first analysis, multiple regression analyses were based on an exhaustive search through all possible combinations of maximally two predictors with a selection criterion of maximally explained variance (program written by H. Winkler). We also made sure that collinearity of predictors was insignificant (or of no concern). This approach avoided problems associated with stepping methods [36]. Restricting predictors to two out of the seven variables for the final model avoided over-fitting of the model. A regression analysis was used for each dependent variable (difference in number of individuals in center, difference in time spent in center) with seven independent variables. Both analyses were repeated with PGLM with the two selected independent variables. For the comparative analyses we employed generalized linear methods [37]. We assumed an evolutionary change that follows a Brownian-motion model [38]. In the case of ‘number of birds in center’ as dependent variable we assumed a Poisson error for the counts. For this analysis 95% confidence intervals and p-values are provided for individual variables. The errors for ‘time spent in center’ followed a normal distribution. Identity was set for the link function in both cases. Residuals were inspected for linearity. Migratory behavior was transformed with square-root and diet change with the square-root-arcsine transform. Experiments in California were conducted earlier than in Mississippi. To exclude a possible influence of season we repeated the analyses with birds from Mississippi only due to the larger data set available.

The experimental design allowed specifically testing for differences in neophobia between resident and migratory populations within some of the species. ANOVA (SPSS 17.0 package) was performed to compare neophobic reactions between resident red-winged blackbirds in California (CA) and resident/migratory red-winged blackbirds in Mississippi (MS) as well as resident (CA) and migratory (MS) populations of Brewer’s blackbirds. Changes in reaction (difference in number of individuals in center, difference in time spent in center) between the first control and experimental trial were compared using site means of each species.

#### Permits

Experiments were conducted under the following permits; Point Reyes National Seashore National Park (Scientific Research and Collection permit PORE-2004-SCI-0032 United Stated Department of the Interior National Park Service), Yazoo National Wildlife Refuge (Special Use Permit No. 04005 US Department of the Interior, Fish and Wildlife Service) and Leroy Percy State Park (verbal agreement by Park Authority (Mississippi Department of Wildlife, Fisheries and Parks). Furthermore, approval was given to conduct experiments on farmland by several farmers. In Mississippi, birds were captured and banded under the Federal Bird Banding permit (US Department of the Interior) No. 09613 issued to Paul B. Hamel.

## Results

### Overall Response to Novelty

**Figure 1:**
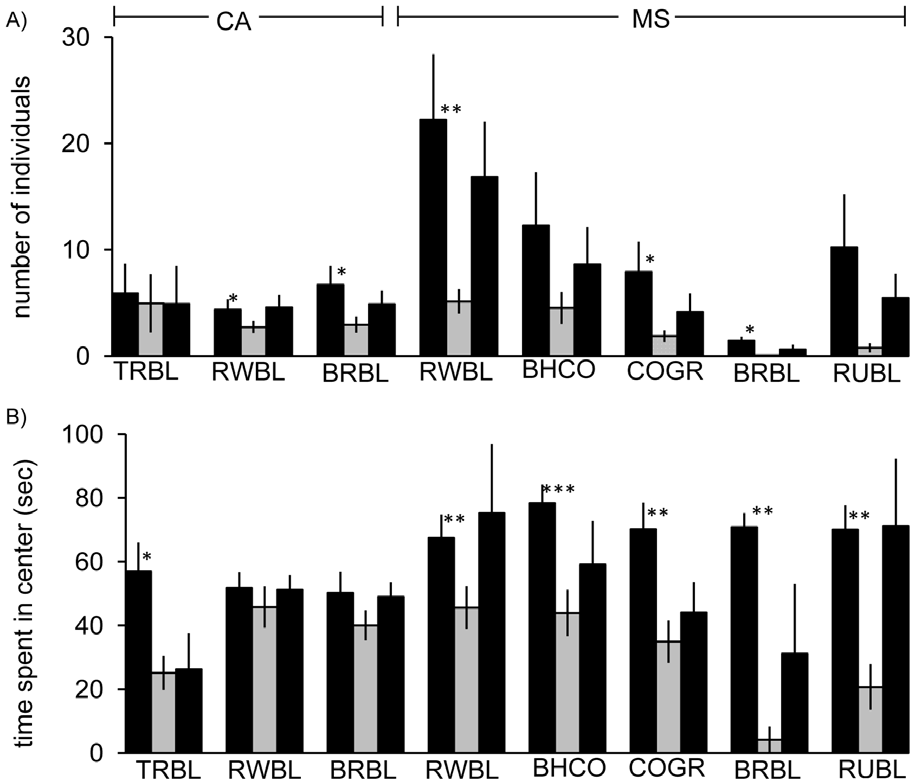
Neophobia reactions of the species under investigation. Means and standard errors of A) number of individuals per species in center and B) time spent in center are shown for the two control and the experimental trials. Stars indicate significant differences between control trial 1 and experimental trial (paired t-test). *: p≤0.05; **p≤0.01 Black bars: control trials, grey bars: experimental trial TRBL: Tricolored blackbird, RWBL: Red-winged-blackbird, BRBL: Brewer’s blackbird, BHCO: Brown-headed cowbird, COGR: Common grackle, RUBL: Rusty blackbird; CA: California; MS: Mississippi.

We tested for the influence of novel objects placed around the feeding plot. The number of individuals in center differed significantly between trial types (F_2,14_ = 78.7, p<0.001) with fewer individuals in center during experimental trials (Fig. 1a). The interaction between trial type and species was significant (F_2,14_ = 2.8, p<0.005), indicating a differential response of species to the novel objects. The number of individuals present in center did not vary significantly between species when considering all trials (F_1,7_ = 1.8, p>0.05). The time spent in center varied significantly between control and experimental trials (F_2,14_ = 16.1, p<0.001) with shorter times in center in experimental trials (Fig. 1b). The interaction between trial type and species was not significant (F_2,14_ = 1.2, p>0.05) nor was the effect of species alone across all trials (F_1,7_ = 1.5, p>0.05).

Results of both tests indicate that the novel objects elicited a neophobic response rather than the response being the result of satiation as number of birds and time spent in center increased again during the second control trial (Fig. 1).

### The Role of Ecological Variables in Explaining Neophobic Reactions

**Figure 2:**
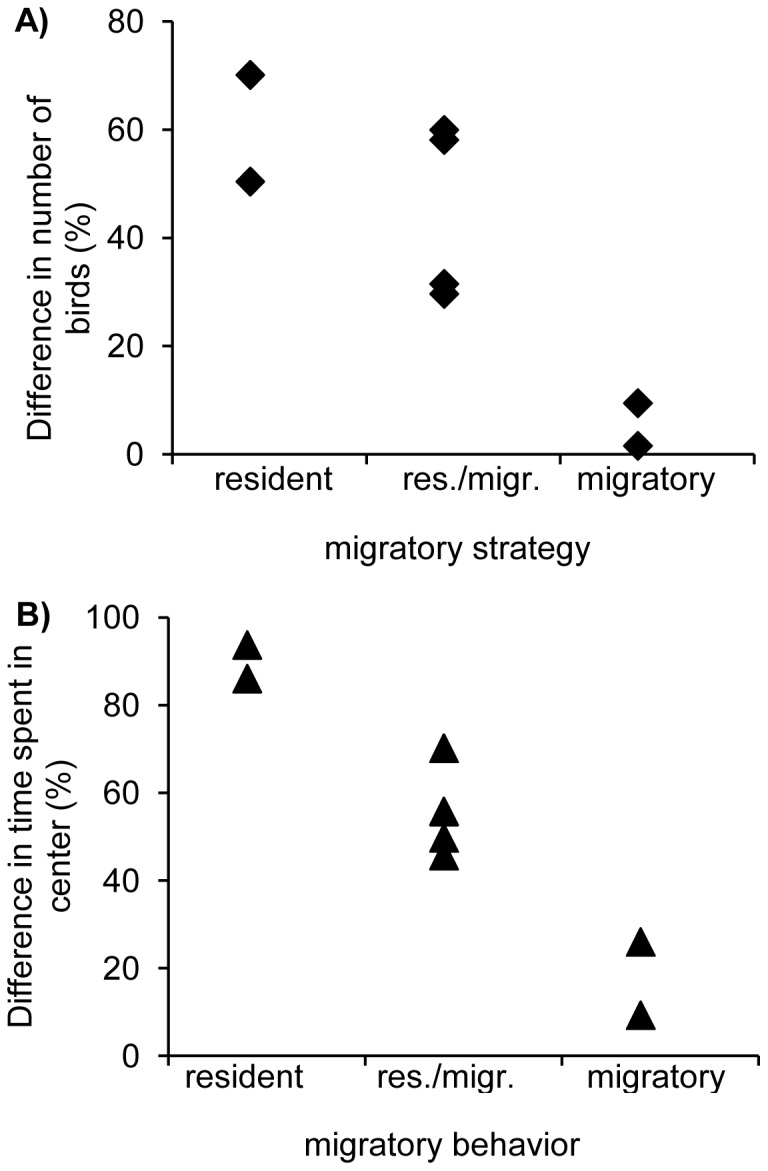
Relationship between neophobia reactions and migratory strategy. The difference in (A) number of individuals in center and (B) the difference in time spent in center between the first control and experimental trial (expressed as percent in relation to the control trial) are plotted against migratory strategy of the species. Res./migr.: resident/migratory.

Whether neophobia reactions were related to ecological plasticity or migratory strategy was first tested with species considered as independent data points. Differences in number of individuals in center between the first control and the experimental trial were significantly related to migratory strategy (95% confidence intervals: −189.31; −97.18, p<0.0001) and marginally significant for diet breadth (−6.85; 0.43, p = 0.035). The overall model was highly significant (chi^2^ = 108.2, df = 2, p<0.00001). During the experimental trial, the number of individuals in center dropped to less than 10% of the control trial in migrants, whereas in the residents, numbers dropped to maximally 50% of the control trial (Fig. 2a). Populations consisting of residents and migrants ranged in between. Diet breadth was negatively related to neophobic reactions with fewer individuals of diet generalists returning to the feeding plot when novel objects were present. Likewise, differences in time spent in center between the first control and the experimental trial showed a significant relationship to migratory strategy (t = −11.457, p<0.00009) and the overall number of birds present (t = 3.189, p = 0.024). Overall, the two variables explained 97% of the variance (regression analyses; F_2,5_ = 77.2, p<0.0002). Migrants reduced the time spent in center by at least 74% in comparison to the control situation, whereas residents did so by only 14% (Fig. 2b). Residents/migrants were again in between these two extremes. Furthermore, the more individuals of a species were present around the foraging site the less they reduced their foraging time when objects were present, i.e. the weaker their neophobic response. When conducting the analysis with the difference in time spent in center between the experimental trial and the second control trial results were similar showing a significant relationship to migratory strategy (F_1,6_ = 27.5, p = 0.002) explaining 82.1% of the variance. Considering phylogenetic relationships for differences in reaction between the first control trial and the experimental trial, results changed only slightly for the number of birds in center with both, migratory strategy and diet breadth significant (PGLM:Chi^2^ = 238.3, p<0.00001; 95% confidence intervals migration: −152.39; −138.45, p<0.0001; diet breadth: −5.82; −3.20, p<0.001). For time spent in center, both migratory strategy and overall number of birds present remained significant (r^2^ = 0.93, F2_,5_ = 31.7, p<0.002; 95% confidence intervals migration: −212.10; −176.75, p<0.0001; overall number of birds: 0.48; 1.32, p<0.005).) indicating that species can be considered as independent data points.

Restricting the analyses to the Mississippi region changed results only slightly. Differences in the number of birds visiting the feeding plot were nearly significantly related to migratory strategy (df = 4, r^2^ = 0.72, F_1,3_ = 7.8, p = 0.067), whereas the differences in time spent in center between the first control and the experimental trial showed a significant relationship to migratory strategy (r^2^ = 0.85, F_1,3_ = 16.7, p<0.026).

**Figure 3:**
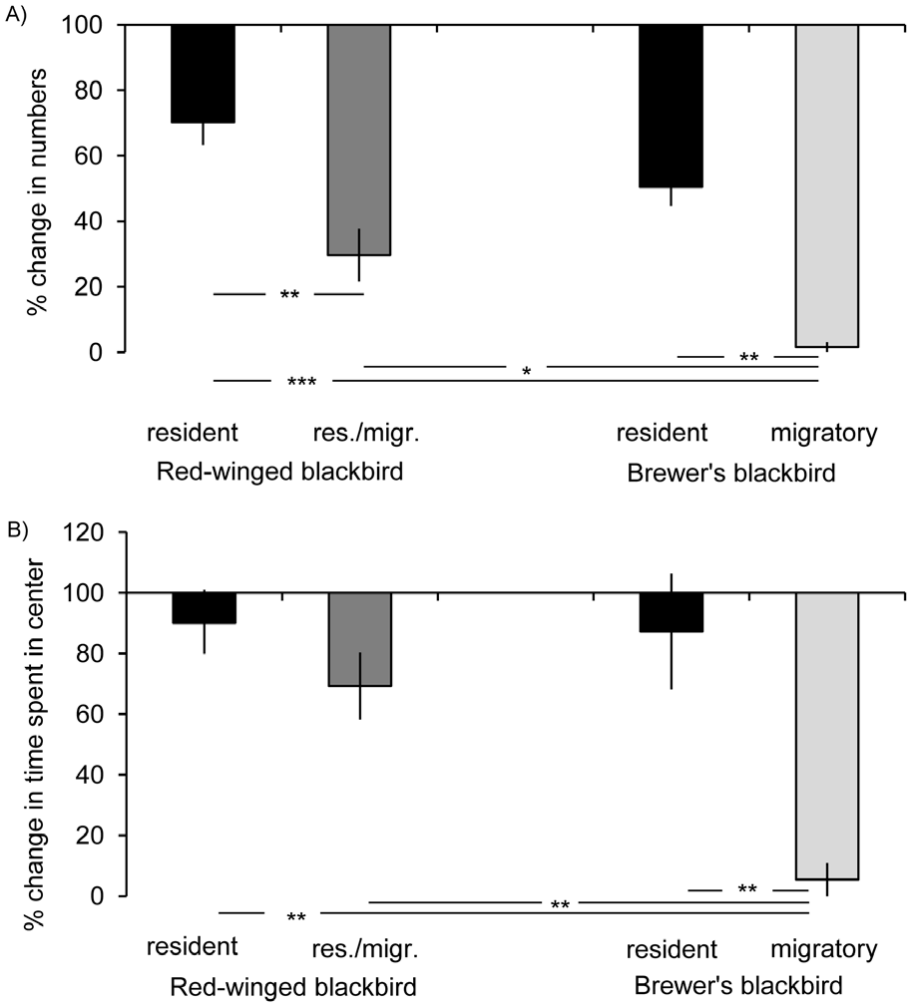
Neophobia reactions within species. Means and standard errors of changes in behavior between control trial 1 and experimental trial are shown for A) number of individuals in center and B) time spent in center for resident and migratory Brewer’s blackbirds and resident and resident/migratory red-winged blackbirds. Changes are given in percent relative to the values during control trial 1 which were set to 100%. Black bars: residents; dark grey bars: resident/migrants; light grey bars: migrants; Res./migr.: resident/migratory; *p<0.05, **p<0.01, ***p<0.001.

The influence of migratory strategy on neophobia reactions was further investigated by a within-species comparison of resident and migratory (resident/migratory in the red-winged blackbird) populations of Brewer’s and red-winged blackbirds. There were significant species differences in the number of birds in center during experimental trials as compared to control trials (ANOVA: F_3,19_ = 9.5, p<0.001). LSD-posthoc tests revealed that during experimental trials significantly fewer individuals of the migratory Brewer’s population in Mississippi returned to the center as compared to the resident Brewer’s and red-winged populations in California and resident/migratory red-winged populations in Mississippi (Fig. 3a). There were no differences in relative numbers of individuals returning to the center between the resident Brewer’s and red-winged populations in California. However, the proportion of individuals staying away from the center during experimental trials was significantly higher in the resident/migratory red-winged population in Mississippi than in the resident red-winged population in California. Species differences were also found in the time spent in center between control and experimental trials (ANOVA: F_3,19_ = 5.0, p = 0.009). Brewer’s population in Mississippi reduced the time spent in center during experimental trials much more than resident Brewer’s and red-winged blackbird populations in California or mixed resident/migratory red-winged populations in Mississippi (Fig. 3b). There were no significant differences in reaction among the resident Brewer’s and red-winged populations in California and resident/migratory red-winged population in Mississippi.

## Discussion

Novel objects placed around a feeding plot elicited a clear neophobic response in all species. However, the strength of reaction differed between species depending on their migratory behavior, diet breadth (number of birds in center) and the overall number of birds present (time spent in center). Migrants showed a much stronger neophobic response than residents. These results also hold when considering only birds from Mississippi although to a lesser extent, possibly because of the reduced sample size and the lower variation in migratory strategy (only pure migratory species and species consisting of resident and migratory populations but no pure residents). Within-species comparisons confirmed different neophobic reactions in relation to migratory behavior. Furthermore, diet generalists tended to be more neophobic than diet specialists with respect to the number of birds feeding with objects and the more individuals of a species were present the less neophobic they were with respect to the time spent in center with objects. Neither body mass nor number of individuals per species present influenced neophobia reactions considering the number of birds returning to the center when objects were present indicating that dominance relationships did not play a role in decision-making whether or not to return. However, how long birds remained in center with objects was affected by group size. Obviously, birds felt safer with more birds present (group protection hypothesis) [39]. Finally, phylogenetic relationships changed results only slightly and even increased the significance of results.

Differences in neophobia can be based on genetic or environmental effects or an interaction between them. Genetic effects are suggested as neophobia is known to have a genetic component [40], [41] and common garden experiments have revealed differences in neophobia between residents and migrants [19] as well as generalists and specialists [13], [14]. However, it has also been shown that experience with enriched or barren environments changes neophobia [42], [13]. Furthermore, results from wild caught and hand-reared sparrows (*Melospiza*) indicate an interaction between genes and environment [13]. In the current study, the contribution of genes and environment could not be separated but the consistency of results across taxa (see below) and geographic locations suggests a stronger genetic rather than environmental effect. Future studies separating the contribution of these two factors on neophobia are needed.

The relationship between migratory behavior and neophobia is in line with our expectation and confirms earlier findings in two closely related old-world warbler species in captivity in which the migratory garden warbler hesitated longer to feed when a novel object was placed beside the feeding dish than the resident Sardinian warbler [19]. Thus, the results from the captive study are confirmed in a natural setting including more species and extend the findings to the number of birds returning and the time spent in close vicinity to the novel object. This generality across taxa and the fact that the warbler study was conducted with hand-raised birds suggest a genetic component of differences in neophobia. Cost-benefit consideration may explain the evolution of different neophobia reactions. Residents may benefit from lower neophobia as even though they may not be bound to their territory during the non-breeding season they are often restricted to a relatively small area throughout their life [43]. Lower neophobia can help getting access to resources before others and ultimately secure residency. Benefits of lower neophobia (earlier exploration and access to resources) may out-weight costs of lower neophobia (predation, injury) in residents as this may allow staying in or near the breeding territory. Migrants, in contrast, may be less bound to a particular area during the non-breeding season (the studied species are all short-distance migrants and stay for relatively short periods on the wintering ground) and may not benefit from lower neophobia in the same way as residents do. Costs of lower neophobia in migrants may therefore, not be out-weighted by higher benefits, while strong neophobia may protect them from possibly dangerous situations. Residents may, therefore, have evolved lower neophobia regarding changes in their familiar environment than migrants.

Besides this genetic component of neophobia, experience may also contribute to different neophobic reactions. Through their year-round residency, residents have a greater familiarity with the area and changes therein (e.g. dangerousness of machinery or bags placed in fields and other habitats) than migrants that stay on the wintering ground for only a few months. Experience with changes in this particular environment during other times of the year may reduce uncertainty in residents through generalization processes [44], [45], [46] and allow adaptation of neophobia reactions to local conditions. Migrants, in contrast, have only a fragmentary knowledge about the wintering ground and possible changes therein. If at all, they can only infer risk from experiences gained somewhere else which may vary greatly between locations and may not allow generalization. Instead of relying on own experience, migrants may use public information [47], [48] by observing resident birds. Migrants are already known to use residents as a cue for breeding habitat selection (heterospecific attraction hypothesis; [49]). They may also use residents to learn about the local risks of an area and stay initially away in potentially dangerous situations.

Migrants have been shown to be less behaviorally flexible (in terms of innovative behaviors) and less successful in invading new sites than residents [50], [51]. Neophobia adds another component that constrains behavioral reactions to environmental change. Firstly, species sensitive to disturbance may have increasingly more difficulties to find undisturbed sites. Secondly, strong neophobic reactions to disturbances negatively influence energy budgets particularly in winter because the birds fly off more frequently and stay away from food sources for a longer period of time. Thirdly, strong neophobia delays exploitation of newly emerging resources. Thus, strong neophobic reactions in migrants may contribute to negative population developments [52] in our increasingly faster changing environment. One of the most neophobic species in our study was the rusty blackbird. It is also the strongest declining songbird in the U.S. which shows long-term as well as acute short-term declines [53], [54]. This decline contrasts sharply with population trends in other blackbird species which have shown tremendous long-term increases and range expansions in the face of anthropogenic habitat change [24], [27].

Despite the overwhelming influence of migratory strategy on the level of neophobia found among different blackbird species, ecological plasticity measured with several variables describing niche breadth still played a role. In our study, diet breadth showed a marginally significant relationship to neophobic reactions when considering species as independent data points and showed a significant relationship to neophobia when considering phylogeny. The weak relationship may be a result of small sample size. Furthermore, information about diet and habitat use was only available on the species level. It is possible, that resident and migratory populations not only differ in their movement patterns but also in their diet and habitat breadth. Finally, there may be an interaction between ecological plasticity and migratory strategy in the sense that for example within each migratory strategy (resident or migratory) ecologically more plastic species are less neophobic than more stereotypic species. Our sample size was too small to test for these interactions. All other studies on ecological plasticity and neophobia were either done exclusively with migrants [10] or residents [7] leaving undetermined a possible influence of interactions between migratory strategy and ecological plasticity on neophobia reactions.In our study a broader food spectrum was linked with higher neophobia regarding number of birds returning to feed when objects were present. In this context it should be mentioned that we measured the reaction to novel objects around a familiar food source (i.e. a change in the environment) rather than reluctance to feed on novel food itself which may lead to different results [4]. Our data support the dangerous niche hypothesis [13] which holds that generalist species that live in dangerous habitats or feed on potentially dangerous foods should show high levels of neophobia indicated by reluctance to approach unfamiliar situations. However, this was obviously not mediated by persecution as we had hypothesized for only habitat breadth but not diet breadth was related to persecution in our study system. It therefore, seems that species which can utilize a variety of diets are more neophobic than more specialized species which may be a protective mechanism [13]. This is the first study that provides some support to the dangerous niche hypothesis in a wild population which is not related to persecution. However, more data are needed to confirm this result.

To summarize, blackbird species that differed in their ecological plasticity and migratory behavior reacted differently to changes in their environment. The strength of the neophobic reaction varied with the migratory strategy. Genetic as well as environmental factors (experience) may explain these differences. The results indicate that environmental changes may be particularly critical for migrants. More research is needed to understand better the relationship between ecological plasticity and neophobic reactions.
